# Advances in the Mitigation of Microbiologically Influenced Concrete Corrosion: A Snapshot

**DOI:** 10.3390/ma17235846

**Published:** 2024-11-28

**Authors:** Husnu Gerengi, Ertugrul Kaya, Moses M. Solomon, Matthew Snape, Andrea Koerdt

**Affiliations:** 1 Corrosion Research Laboratory, Department of Mechanical Engineering, Faculty of Engineering, Düzce University, 81620 Duzce, Türkiye; 2 3-S Engineering Consultation Industry and Commerce Incorporated Company, R&D Centre, 81620 Duzce, Türkiye; 3 Department of Chemical and Environmental Engineering, University of Nottingham Ningbo China, Ningbo 315104, China; 4 SGS MIRAS Consultancy Services, Global Biosciences Centre, 1600-604 Lisbon, Portugal; 5 Bundesanstalt für Materialforschung und Prüfung (BAM), Unter den Eichen 87, 12205 Berlin, Germany

**Keywords:** microbiologically influenced corrosion, concrete, concrete corrosion, microbiome-inhibiting

## Abstract

Concrete, a versatile construction material, faces pervasive deterioration due to microbiologically influenced corrosion (MIC) in various applications, including sewer systems, marine engineering, and buildings. MIC is initiated by microbial activities such as involving sulfate-reducing bacteria (SRB), sulfur-oxidizing bacteria (SOB), etc., producing corrosive substances like sulfuric acid. This process significantly impacts structures, causing economic losses and environmental concerns. Despite over a century of research, MIC remains a debated issue, lacking standardized assessment methods. Microorganisms contribute to concrete degradation through physical and chemical means. In the oil and gas industry, SRB and SOB activities may adversely affect concrete in offshore platforms. MIC challenges also arise in cooling water systems and civil infrastructures, impacting concrete surfaces. Sewer systems experience biogenic corrosion, primarily driven by SRB activities, leading to concrete deterioration. Mitigation traditionally involves the use of biocides and surface coatings, but their long-term effectiveness and environmental impact are questionable. Nowadays, it is important to design more eco-friendly mitigation products. The microbial-influenced carbonate precipitation is one of the green techniques and involves incorporating beneficial bacteria with antibacterial activity into cementitious materials to prevent the growth and the formation of a community that contains species that are pathogenic or may be responsible for MIC. These innovative strategies present promising avenues for addressing MIC challenges and preserving the integrity of concrete structures. This review provides a snapshot of the MIC in various areas and mitigation measures, excluding underlying mechanisms and broader influencing factors.

## 1. Introduction

Concrete is versatile in application, especially as a construction material. It finds applications in sewer systems, offshore installations, marine engineering, and in buildings.

However, in most of these application areas, concrete structures suffer deterioration occasioned by microbial activities [1]. MIC begins when microorganisms naturally present on concrete structures engage in biochemical activities that might affect the structures by influencing protective surface films and thereby creating corrosive conditions or producing thick deposits. For instance, in sewer and marine systems, MIC is a common problem [2] and the failure of concrete structures in these systems causes vast economic losses [3]. This process starts usually with sulfate-reducing bacteria (SRB) converting sulfate to hydrogen sulfide in anaerobic environments. Then, sulfur-oxidizing bacteria (SOB), especially those belonging to the genus Thiobacillus, convert hydrogen sulfide into corrosive sulfuric acid [4]. Some fungi can be also involved in this process [5]. In marine concrete engineering, structures located in tidal and splash zones face deterioration, predominantly attributed to *Pseudoalteromonas*. Others contributing to damage in these areas include members of species belonging, besides others, to the species *Vibrio*, *Pseudomonas*, and *Arthrobacters* [6]. The degradation of concrete in irrigation and hydroelectric canals, the emergence of spots or patches on concrete walls and the biological decay of mortars on building facades are frequently attributed to the proliferation of algae and cyanobacteria. Algae require light for photosynthesis, which restricts them to photic zones—the upper layers of water where sunlight penetration supports their growth. Consequently, the proliferation of algae on concrete surfaces, such as the walls of water storage and conveyance structures, does not contradict this requirement, as these surfaces effectively act as artificial photic zones by receiving sufficient light [6,7,8].

The sewer system is home to a microbial ecosystem that is under the influence of various environmental factors. Similarly, the corrosion of concrete can be frequently affected by multiple environmental variables, including high salinity, high temperature, etc. [9]. Some microorganisms produce, under anaerobic conditions, acidic metabolites that are secreted into the environment and potentially cause corrosion and thereby increase the porosity of the concrete [9]. Consequently, approximately 40% of concrete failures in sewerage systems have been reported to be due to MIC [10].

While there is no official data detailing the total expenditure associated with MIC, it is estimated that it constitutes >50% of underground pipeline failures [11]. The repair and maintenance of both private and public sewerage systems in Germany is estimated to be approximately USD 40 billion [1]. In particular, 40% of this expenditure is allocated to damage caused by MIC-influenced corrosion [1]. MIC causes significant economic losses in the USA, exemplified by rehabilitation costs estimated at USD 390 billion over approximately 20 years [4]. The annual rehabilitation costs in Germany and the UK are also estimated at € 450 million and £ 85 million, respectively [12]. Beyond the economic impact, the increasing focus on MIC is driven by growing concerns about health and environmental issues, as evidenced by the growing interest in these issues [13].

The complexity of the issue becomes apparent when one considers that, even after more than a century of research, the MIC of concrete is still an ongoing topic of debate. In addition to the existing challenges, new changes in our society must also be considered. The problem caused by microbiological activity, which has been present in wastewater systems for decades, is being greatly exacerbated by water conservation measures. It is important to recognize that, despite significant efforts, current research results have not reached a level that can significantly influence current construction practices [14]. Furthermore, there is a lack of universally recognized standardized test methods to comprehensively assess the problem, as evidenced in references such as [14,15,16,17]. This fact not only adds another layer of complexity but also limits the understanding of MIC in the concrete sector, and seems to have accelerated dramatically since the reduction in water consumption. Since the corrosion of concrete in the wastewater sector is a dynamic process, measuring electrochemical parameters online could provide more accurate results, and dynamic electrochemical impedance spectroscopy [18], which determines the instantaneous corrosion mechanism, can greatly facilitate the understanding of future MIC studies.

Based on the impact that MIC has on concrete assets, as well as the challenges in conclusively assessing MIC that were described above, this study confines its scope to a comprehensive review and discussion of assessment methods for the MIC of concrete and potential future mitigation measures. While recognizing the significance of other key elements such as the underlying mechanisms, crucial influencing factors, and observations from field and laboratory studies, these aspects fall beyond the current study’s purview. An effort has been made, nonetheless, to shed light on the intricate relationship between microbiological activities, environmental conditions, and the corrosion of concrete materials.

## 2. Microbially Influenced Concrete Corrosion

### 2.1. The Part Played by Microorganisms

**Figure 1:**
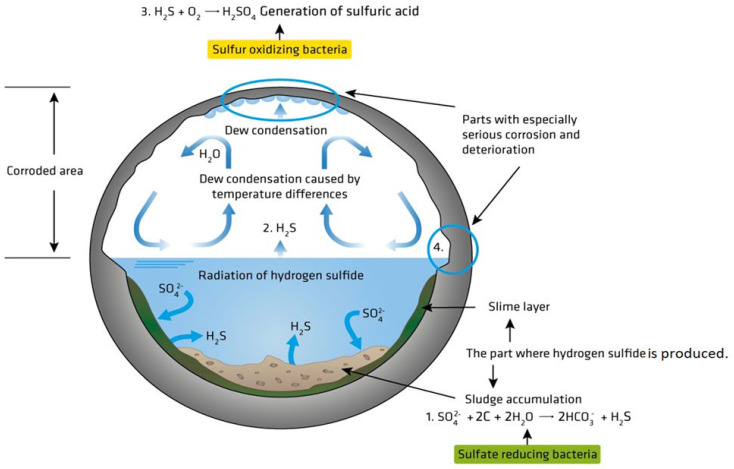
The schematic illustration of the primary events associated with the MIC of concrete when exposed to sewer environments. Reproduced with permission from Wu et al. [19]. © 2019 Elsevier Ltd., Amsterdam, The Netherlands.

The activities of microorganisms can affect the physical, aesthetic, and chemical properties of concretes. Microbial growth or movement on concrete structures could lead to physical or mechanical breakdown. Fouling due to biofilm formation can cause aesthetic issues, and chemical deterioration can occur due to the excretion of metabolites or corrosive substances, such as H_2_S, H_2_SO_4_, etc., by microorganisms. These substances negatively affect the structural properties of the material, causing increased porosity and the weakening of the mineral matrix in the concrete structure. Figure 1 illustrates the primary events associated with the MIC of concrete in a typical sewer environment.

Reproducing MIC in a laboratory setting is challenging due to the inability to adequately mimic the synergistic interactions between the multiple types of microorganisms potentially involved in the process (i.e., bacteria, algae, and fungi). In addition, small but important changes in the environment cannot be simulated in the lab. The number of possible changes in parameters is high, and therefore, monitoring the situation/parameter present in the field would make more sense to detect if MIC is the reason for damage or other circumstances. Consequently, due to the lack of access of researchers to communities from real MIC cases, MIC-related laboratory experiments are mainly limited to pure or defined cultures [20]. Furthermore, studying concrete MIC in diverse systems entails an in-depth understanding of the microorganisms involved in the process and their metabolism. This section provides an overview of the current understanding of the role of microorganisms in the biodeterioration of concrete infrastructure, including a discussion of the possible mechanisms involved in biodeterioration.

#### 2.1.1. Oil and Gas Industry

In offshore platforms, reinforced concrete stands out as the predominant structural material, primarily because of its affordability, outstanding hydrophobicity, and ease of fabrication into various sizes and shapes. Its alkaline nature results in the formation of a passive film, typically FeO, on steel, providing effective corrosion resistance. This quality has led to its widespread use in safeguarding metallic pipelines. It is common to see oil and gas pipelines buried in concrete that serves as a protective external cover. Finally, several oil and gas facilities have used concrete-supported gravity-based storage (GBS) tanks for crude oil inventories before export. Nevertheless, the microorganisms in the environment can significantly impact the efficacy of the concrete due to the creation of aggressive environments through factors such as influencing oxygen concentration cells, altering pH, or producing corrosive metabolites. These microbial activities pose challenges to the long-term durability and corrosion resistance of concrete structures in such environments [19,21].

**Figure 2:**
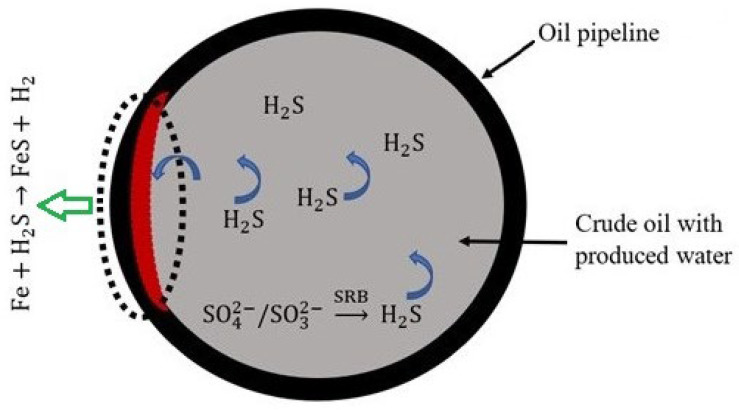
The schematic diagram of microbial corrosion of oil pipelines by the metabolic activities of microorganisms in the environment. Reproduced from Chen et al. [22]. © 2013 Chen et al.; licensee Chemistry Central Ltd., Mountain View, CA, USA.

Similar to the chemical deterioration observed in concrete sewer pipes due to the activity of SOB, the activities of SRB can substantially damage concrete employed in oil storage tanks and the support abutments of offshore structures. Bulk storage tanks for ferrous sulfate repository (a.k.a., sulfate storage tanks) encourage the growth of SRB. Changes in O_2_ concentration in the H_2_O phase of storage systems can also encourage the interaction of SRB and SOB. Such a relationship results in the formation of acidic metabolites [22,23]. Furthermore, the mutualistic symbiosis between aerobic oil-oxidizing bacteria (OOB) and SRB has been associated with the degradation of concrete components in oil–water systems [16]. OOB growth and proliferation in crude oil, which occurs before SRB, produces organic acids, predominantly acetic acid which lowers water pH [23]. The growth of SRB aided by crude oil and its by-products produces H_2_S, which, upon oxidization (the dotted part of Figure 2), generates H_2_SO_4_ (Figure 2) [7,22,23].

Most times, the contact of concrete with acidic compounds produces expanded calcium-containing compounds that weaken the concrete and increase the porosity [23]. Beddoe and Dorner [23] observed that, when concrete is attacked by acidic metabolites secreted by microorganisms such as H_2_SO_4_, gypsum precipitates in the corroded layer and reduces the acid penetration but with the expansion and disintegration of the surface concrete occurring. This product causes an increment in the internal pressure of concrete and the potential consequence can be cracking. While concrete corrosion can occur independently of rebar corrosion, it can instigate rebar corrosion by creating conducive conditions. It is a known fact that concrete corrosion lowers concrete’s pH and increases its porosity, both of which contribute to the exposure of reinforcing steel to both chemical and biological corrosion [23].

#### 2.1.2. Cooling Water Systems

The MIC of concrete in cooling towers poses a significant challenge for the geothermal and cooling industry. The conditions prevalent in cooling towers, characterized by highly oxygenated water with temperatures ranging from 27 to 60 °C and a pH ranging from 6 to 9, create an ideal environment for the biofouling of concrete surfaces in contact with H_2_O [24]. The liquid condensate of the make-up water, containing dissolved gases such as H_2_S, NH_4_, and CO_2_ in addition to various dissolved solids, serves as the nutrient-rich medium for microbial growth [25].

**Figure 3:**
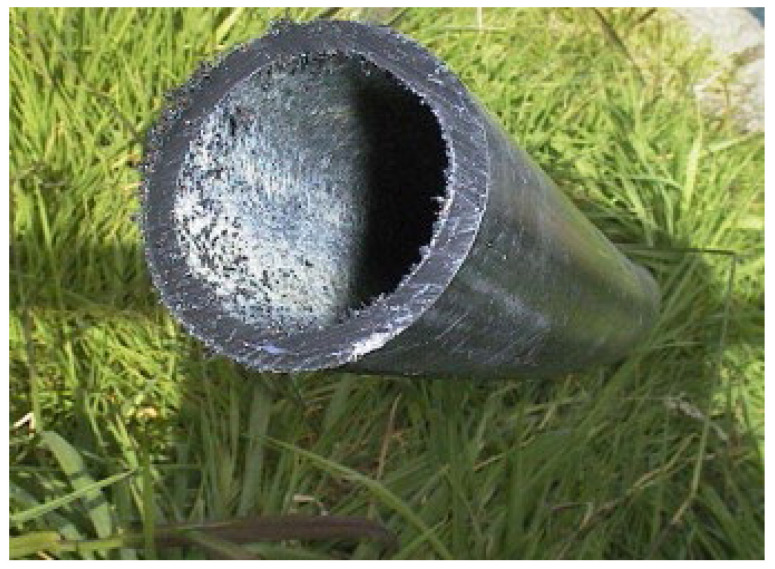
Concrete pipe from a geothermal power plant with an adherent biofilm of sulfur-oxidizing bacteria (white). The Wairakei Power Station is the only geothermal plant in the world with once-through cooling. Shortly after the commissioning of the plant, it was discovered that headspaces in the culverts were suffering from severe acid attacks due to the presence of the oxidation product of H_2_S, i.e., H_2_SO_4_. The pH of the surface moisture was found to be <2, which is common in the presence of “sulfur-oxidizing bacteria” that oxidizes H_2_S to sulfuric acid. Reproduced with permission from Brown and Bacon [25]. © 2008 Elsevier Ltd., Amsterdam, The Netherlands.

Figure 3 depicts a concrete pipe from the bioreactor plant. It shows an attached biofilm composed predominantly of sulfur-oxidizing bacteria (the whitish substances inside the pipe). This biofilm illustrates the presence and activity of microorganisms, particularly those engaged in oxidizing sulfur compounds, in the geothermal power plant environment [25]. The adherence of these microbes to the pipe’s surface is a visible manifestation of microbial processes, potentially influencing the bioreactor’s performance and the overall system dynamics. The key culprits that have been identified to date and seem to be behind the deterioration of cooling towers are typically sulfur-oxidizing and nitrifying bacteria. The breakdown of urea by urobacteria in cooling water produces NH_4_, which can be further oxidized to nitric acid by various bacterial species, including *Nitrosomonas*, *Nitrosococcus*, and *Nitrosolobus*. These metabolic activities induce the biocorrosion of concrete [26].

#### 2.1.3. Civil Infrastructures

**Figure 4:**
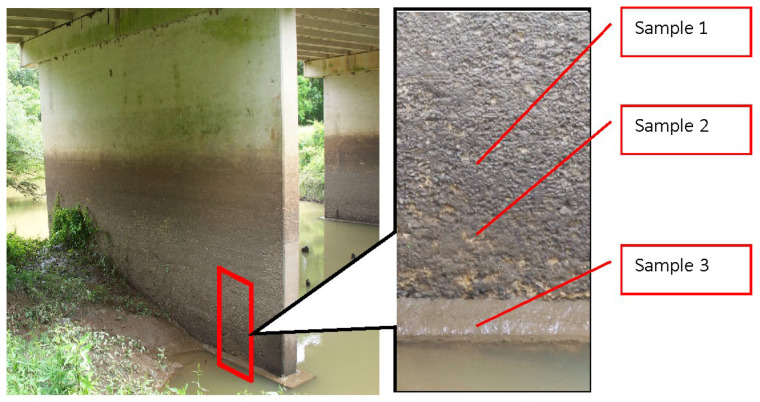
The concrete samples from a bridge site in Texas. Reproduced with permission from Trejo et al. [27].

Microorganisms, while not directly responsible for the deterioration of concrete structures like highway bridges, can significantly undermine the protective or aesthetic surface painting through their metabolic activities. This occurs through the degradation of the surface coating of concrete, allowing moisture and O_2_ to interact with the substrate beneath the concrete surface. Additionally, microbial growth and proliferation on the surface have the potential to substantially diminish the pore alkalinity of the concrete H_2_O and disrupt the passive film surrounding the reinforcing steel [27]. The composition and diversity of microbial communities on concrete surfaces are influenced by factors including pH level, trophic properties (autotrophic or heterotrophic), and nutrient availability [28]. Carbonation contributes to the reduction in alkalinity on the outer surface of reinforced concrete. Deposits from acid rain or air pollution that contain compounds like sulfur and nitrogen can serve as nutrient sources for the proliferation of microorganisms. Examples include sulfur-oxidizing (*Thiobacillus thioparus*, *T. novellus*, *T. neapolitanus*, and *T. intermedia*), nitrifying (thiobacilli), and ammonia-oxidizing bacteria (Nitroso such as *Nitrosomonas* or *Nitrosovibrio*), which can thrive on the surface of reinforced concrete on motorway bridges. Evidence of active sulfur-oxidizing bacteria was found in concrete samples from a bridge site in Texas (Figure 4) [27].

To characterize the microbial communities on the bridge site, several concrete specimens with mild to severe surface deformation above the water table were scrutinized. The findings revealed a swift weight loss, reaching 5.7% within 3 months, in contrast to a mere 0.3% weight loss observed in non-inoculated control samples. Additionally, *Streptomyces* sp., which demonstrated the ability to oxidize thiosulfate to H_2_SO_4_, was isolated from the corroded specimens of the corroded bridge structure [27].

**Figure 5:**
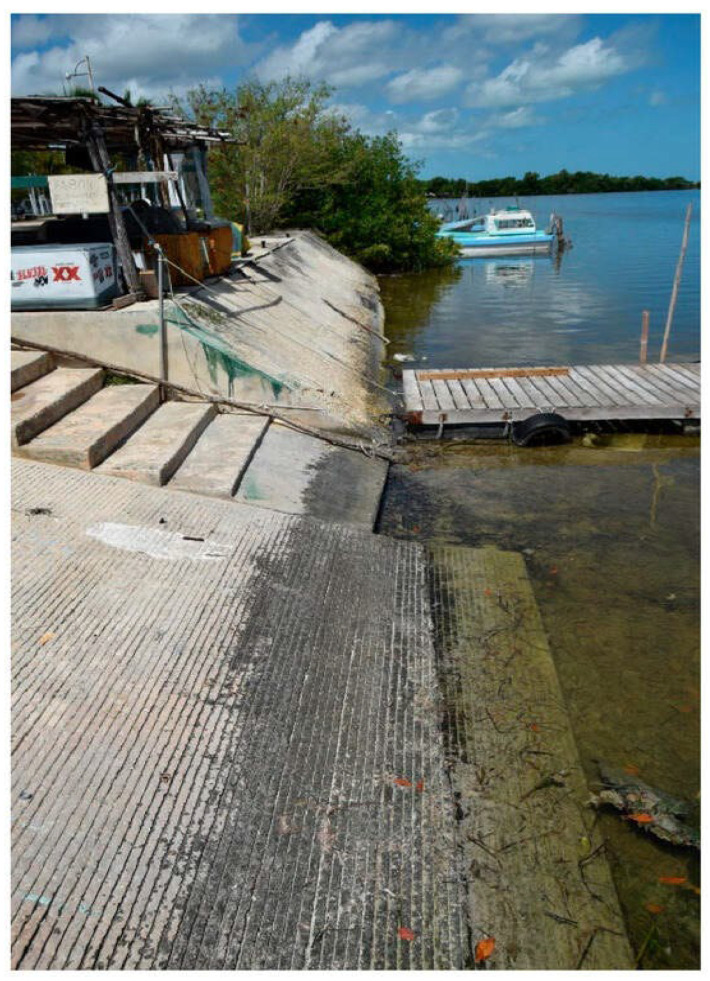
Concrete docks for artisanal fishing boats in Campeche, southern Mexico, are an example of microbial colonization patterns. Reproduced from Gaylarde and Ortega-Morales [30]. © 2023 by the authors. Licensee: MDPI, Basel, Switzerland.

In submerged zones, concrete permanently immersed in water can avoid strong corrosion if oxygen levels in seawater are low enough. However, microbes are everywhere, and those active at low oxygen levels can still produce corrosive compounds [29]. It should be noted that, in shallower waters, sufficient oxygen may be available, and biological activity, with its associated hazards, will certainly continue. Figure 5 shows the slope of the concrete on a small quay; the black biofilm in the splash zone is quite different from the green zone containing photosynthetic cyanobacteria and algae [30].

#### 2.1.4. Sewer Systems

**Figure 6:**
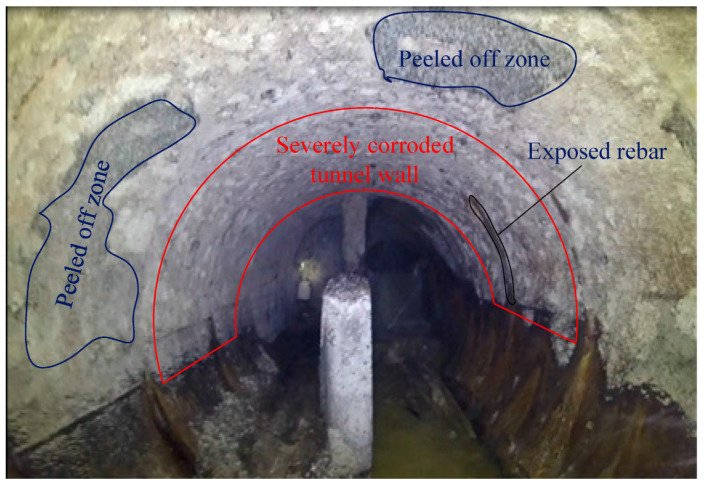
Corrosion conditions in a chamber located under 127 Street and 153 Avenue in Edmonton, Canada. Reproduced from Wu et al. [34]. © 2018 by the authors. Licensee: MDPI, Basel, Switzerland.

The biogenic corrosion of concrete pipes in sewer systems has been widely studied. As earlier explained, sewer’s biogenic corrosion usually begins with the bio-reduction of sulfate and organic sulfur to H_2_S by SRB such as *Desulfovibrio* sp. and occurs under anaerobic conditions [31]. The initial stage of MIC in concrete is abiotically driven. The acidic gases in the sewer headspace (e.g., CO_2_) reach the moisture condensing on the concrete surface. As a result, the pH on the concrete surface decreases from values around 13 down to around 9. Concurrently, the pH on the surface of the concrete is further decreased by the H_2_S also present in the sewer headspace, neutralizing the formerly alkaline surface of the concrete pipe [4]. The decline in pH instigates the growth of SOB, which enter into the development stage. The activity of SOB such as the conversion of H_2_S to H_2_SO_4_ contributes to concrete deterioration [11,31]. The reaction of H_2_SO_4_ with Ca(OH)_2_ in concrete leads to the production of expansive calcium-based sulfates and silicates, such as calcium sulfate dihydrate (CaSO_4_·2H_2_O), calcium silicate Ca_3_Si(CO_3_)(SO_4_)(OH)_6_·12H_2_O), or hydrous calcium aluminum sulfate (CaO_3_·Al_2_O_3_·(CaSO_4_)_3_·32H_2_O) [32]. The production of these expanding products causes internal stresses in the structure, and this can lead to concrete cracking (Figure 6). Consequently, the formation of an additional surface area caused by the expansion of calcium-based sulfates and silicates provides additional sites for microbial corrosion to take place. Additionally, such expansion weakens the structural integrity of concrete, shortening the lifespan of the pipe [33].

**Figure 7:**
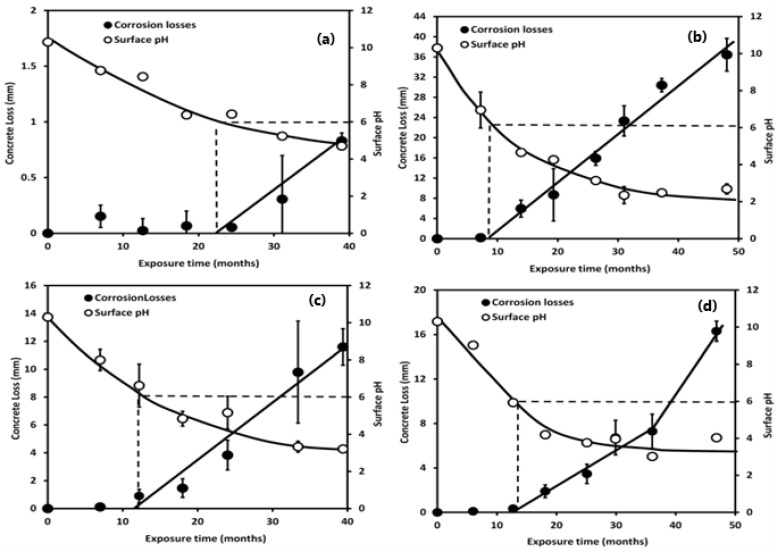
Corrosion losses and surface pH trends for concrete samples from (**a**) Melbourne A, (**b**) Perth A, (**c**) Melbourne B, and (**d**) Perth B sites. The dotted line shows that the onset of corrosion losses coincides with the point at which the surface pH is reduced to 6, irrespective of the aggressiveness of the site. Reproduced with permission from Wells and Melchers [41]. © 2015 Published by Elsevier Ltd., Amsterdam, The Netherlands.

The colonization of sulfate-reducing bacteria, thiosulphate-reducing bacteria (TRB), and sulfur-oxidizing bacteria on the surface of sewer pipes is closely tied to the sulfur cycle [35]. The initial colonization of the pipe crown surface is observed when *Thiobacillus thioparus* reduces the pH to 9 [35]. The oxidation of thiosulfate by *T. thioparus* triggers various reactions involving polythionic acids, leading to a further decrease in pH to levels below neutrality. However, as the degradation process advances, *T. thioparus* gradually diminishes. When the pH of the concrete surface drops to moderately acidic conditions and thiosulfate is present as a nutrient source, species such as *Thiobacillus novellus*, *Thiobacillus neopolitanus*, and *Thiobacillus intermedius* begin to colonize the surface of the asset, contributing to a further drop in pH. As pH values fall below 5, the rapid abiotic or biological conversion of thiosulfate to elemental sulfur occurs, leading to the robust colonization and growth of *Acidithiobacillus thiooxidans* [35,36]. Despite the significant role of *A. thiooxidans* in biological degradation, the microbial community’s composition can vary widely in different sewage systems. Various microorganisms, including *H. neapolitanus*, *T. intermedia*, *T. plumbophilus*, *A. thiooxidans*, *A. acidophilum*, *L. ferrooxidans*, *T. ferrooxidans*, *A. mycobacterium*, *Sphingobacteriales*, *Xanthomonadales*, *Methylacidiphilum*, and *Mycobacterium*, have been isolated from corroded concrete samples [35,36,37,38,39,40]. Wells and Melchers [41] observed that, in six different sewer lines across Australia, corrosion losses coincided with the point at which the surface pH was reduced to 6 (Figure 7). The point where the rapid growth of *A. thiooxidans* follows a transitional phase that initiates concrete deterioration is crucial. Wells and Melchers also noted that the loss in concrete mass was affected by time. The time required to reach the pH level at which corrosion occurs, depending on the local conditions, can vary from several months to several years. Besides pH and time, concrete loss rates have been reported to be affected by sewer gas temperature, relative humidity, aeration rate (dissolved oxygen levels), and H_2_S dosage [41].

### 2.2. Strategies for MIC Mitigation in Concrete

As opined earlier, MIC is caused by the metabolic activities of microorganisms; hence, mitigation strategies are usually focused on either inhibiting the biotic factors or the abiotic factors, and sometimes, the synergistic effect of both the biotic and abiotic factors are used. Many strategies have been explored for controlling MIC, such as inhibitors and biocides, but the toxicity and harm resulting from the chemical composition of these compounds have been of great concern to the environment.

#### 2.2.1. Coatings and Linings

**Figure 8:**
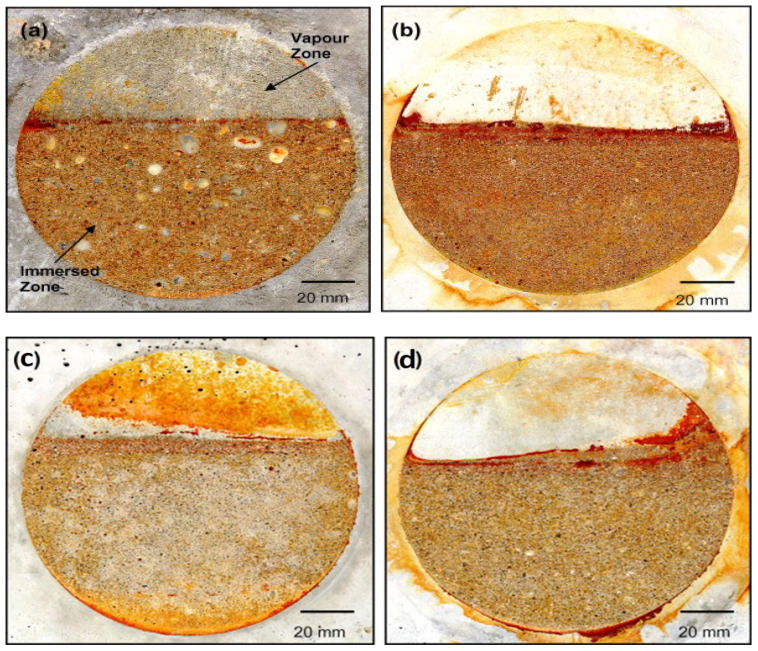
Appearance of (**a**) ASTM C150 Type V (sulfate-resistant) concrete [43], (**b**) 40% blast furnace slag concrete, (**c**) 5% silica fume concrete, and (**d**) 10% silica fume concrete after exposure to *Thiobacillus ferrooxidans*. The concrete in (**a**,**b**) shows etching and staining in the immersed zone, while the ones in (**c**,**d**) show slight etching in the immersed zone. Reproduced with permission from Berndt [43]. © 2011 Elsevier Ltd., Amsterdam, The Netherlands.

Surface coatings are the most commonly adopted MIC mitigation strategies. Coatings are usually chemical-based materials applied physically (physicochemical methods) on the surface of concrete, creating a protective layer on the surface and pores of concrete to inhibit the action of microbes and microbial-generated corrosive compounds. The non-biodegradability of synthetic polymers has proved functional in their microbial resistance and has found use in concrete coatings. These coatings usually contain polymers such as epoxy, polyurea, polyester, fluorine, silicon, and fiberglass that are less destructive to the environment and are efficient in blocking the impact/permeability of gases or aqueous acids [42]. The use of epoxy-based coatings has specifically been discovered to provide an excellent protection from SOB (*Thiobacillus ferrooxidans*) (Figure 8), although limited by the industrially recommended coating thickness, for a minimum period of 60 days [43]. The unique properties of epoxy resin, such as exceptional adhesion to several materials, high strength, and strong resistance to chemicals [44], make it an excellent coating option against MIC. The excellent bonding properties with other materials, such as fiberglass, polyurethane, and metallic oxides, have improved its corrosion resistance abilities. A study by Almusallam et al. [45] discovered epoxy’s special resistance against wetness and chemicals in concrete following a study that revealed little or no deterioration after 60 days of exposure to sulfuric acid. Also, combining epoxy with metal oxides, such as silver and copper, could reduce the action of MIC up to 100% and 92%, respectively, for up to 26 days with little or no formation of any slime layer [46]. More recent studies have confirmed the strong antimicrobial activity of graphene oxide [47] and graphite in combination with metal oxide [48], titanium oxide, aluminum oxide, and a number of other metal oxides [49,50,51], thus making a potent combination with epoxy for MIC mitigation.

Polyurethane composites have also gained traction for their resistance to MIC. Polyurethane, a versatile plastic, is commonly used in a variety of industrial and consumer applications because of its unique physiochemical properties, such as biostability and biocompatibility [52]. Polyurethane is usually modified with special materials, such as water scavengers, surfactants, and metal nanoparticles, which provide acid, base, water, or microbial resistance in those environments [52,53,54]. Polyurethane coatings have shown that they can extend concrete life up to 57 times compared to uncoated concrete without any failure in over 5 years of extreme sulfuric acid immersion [55]. Compared to other polymers used, like epoxy, acrylic, or even chlorinated rubber, polyurethane has superior performance against chloride-based corrosion, improving reinforced concrete life by up to 30 times compared to uncoated concrete [45].

Sulfate-reducing microorganisms (SRMs), such as *Thermococcus*, *Thermotoga* spp., or *Dethiosulfovibrio peptidovorans*, have been identified in oil and gas as MIC accelerators through a process called “cathodic depolarization”, which is basically the electrochemically produced hydrogen consumption by microbes [56]. They are prevalent in oil and gas pipelines, offshore platforms, and storage tanks as a result of the use of cheaper, flexible, and wear-resistant reinforced concrete [1]. However, the microbes (heterotrophic) [55] in petroleum reservoirs have been classified as relatively redundant due to the depth of operations and the temperature at those depths in these reservoirs (>80 °C) and its related low available energy, nutrients, and organic carbon [57]. In spite of this, high-density polyethene (HDPE) has been identified as a viable alternative to line short sections of piping within reinforced concrete. Other liners/coatings that have been used include polyvinyl chloride (PVC), acrylic, cement mortar, and tars [43,45,58].

#### 2.2.2. Biocides

In MICs, biocides are at the forefront of mitigation strategies [59,60]. The term “biocides” describes a wide range of chemicals that are used to control and prevent the negative effects of different microorganisms. These mitigants work by reducing the formation of biofilms on the metal surface. As in other mitigation methods, the biocide chemicals and composition used are dependent on the particular microorganism, the environmental conditions, and the restrictions. However, Skovhus et al. [60] suggested that, rather than generalization, the selection of an appropriate biocide should be carried out on-site, with pipeline fluids, and focused on the immobile microorganism’s control. The metals used in reinforced concrete production include stainless steel, mild steel, galvanized steel, carbon steel, and other “rebars”. Carbon steel is a cheaper option, and it has been used in many applications but is susceptible to corrosion because of the low amount of chromium. Stainless steel, on the other hand, has a higher MIC resistance because of the formation of a chromium oxide layer between the steel and the concrete [59], leading to higher costs. However, due to the lower cost of carbon steel and the alloy’s rigidity, the use of biocides in combination with metal oxides (inhibitor formulations) has been suggested to increase MIC resistance. Also, high-carbon steels have been suggested for their good MIC resistance against *Desulfovibrioalaskensis* in seawater structures [61,62]. Titanium oxide has performed excellently well as an inhibitor/resistor in combination with biocides [62,63]. However, the cost of titanium hinders its application to small-scale uses. Other oxides, such as nickel, copper, and aluminum, are potent alternatives, although not as effective as oxides of titanium, and are specific to the MIC system. Cerium, in particular, has been discovered as potent enough for biocide inhibitors but lacks practical implementation [59]. Glutaraldehyde is the most commonly used biocide in the industry.

In the oil and gas industry, oxidizing and non-oxidizing biocides have been used to control the action of microbes by releasing radicals and consuming sulfates [64]. Non-oxidizing biocides are, however, preferred because of their multifaceted utilization along with their biodegradability and cost efficiency. Oxidizing biocides like chlorine, hydrogen peroxide, magnesium, and sodium hydroxides, react faster than the non-oxidizing biocides but could be corrosive to metals; hence, they are restricted to a more long-lasting microbial control like seawater-enhanced oil recovery procedure [60]. Popular non-oxidizing biocides that can be used in oil and gas well cementing structures are glutaraldehyde and Tetrakis hydroxymethyl phosphonium sulfate (THPS); both are industry-specific non-oxidizing biocides. They are sulfide scavengers and can crosslink amino groups in the cell wall but are inactive to amine [60,64,65]. For carbon steel usage, TPHS, triazines, isothiazolinone, and organobromines have found usage in oil and gas infrastructures.

However, biocide usage has been termed insufficient as a mitigation method in MIC because many biofilms develop resistance to these biocides after multiple and long usages [55]. Also, some chemicals, such as sodium bisulfite, an oxygen scavenger, can react with these biocides (glutaraldehyde), thereby inhibiting its function as a biocide [66]. Some other biocides are not environmentally friendly and need to be used in combination with other chemicals for efficient application. The use of a hybrid concrete mixture has been suggested to supplement the use of biocides and create a synergetic antimicrobial corrosion effect [1]. A hybrid concrete mixture is a concrete where two or more materials are added to concrete to enhance its properties.

#### 2.2.3. Hybrid Concrete Mixtures

Concrete has been modified in several ways to fight MIC. These modifications extend beyond the mere use of coatings/linings, as discussed previously, and include the mixing of polymer materials like plastic fine aggregates manufactured from electronic waste [67], polyethene terephthalate (PET) [68], acrylic [1], HDPE [69], LDPE [70], polypropylene (PP) [71,72], or styrene-acrylic ester [70] with concrete. The primary objective of incorporating polymers into the concrete matrix is to decrease its permeability and slow down the infiltration of corrodents, such as hydrogen sulfide and carbon dioxide, and the entry of microorganisms and their byproducts. The decreased permeability of polymer-modified concrete is a result of the bridging of microcracks, the reduction in pore size, and the blockage of pores by polymer particles.

PET has shown excellent resistance against sulfuric attacks, especially under crushing loads. The response of PET to crushing loads was similar to that of ultrasonic waves [68]. PET and PP-based concrete also show higher compressive, tensile, and flexural strength than conventional concrete, which are beneficial for concrete structures against permeability. However, the excessive addition of polymer (beyond 15–20% [73]) can hamper compressive strength and cause retarded cement hydration; hence, it is important to use optimal polymer loadings [69,70]. However, polypropylene and polyethene compounds have been identified as vulnerable to some strong acids and even oxygen and would require antioxidants to maintain their anticorrosion abilities in concrete. The use of chlorinated polyvinyl chloride has demonstrated resistance to all environmental factors, including the combination of oxygen and water, pollutants, ozone, salt air, and bacteria, which can cause MIC in metals used in reinforced concrete [74]. Generally, the use of polymer plastics as MIC retardants for these hybrid concrete has remained a competent candidate owing to the dual effect on reduced plastic waste disposal and effects on concrete impermeability.

Just as in coatings and linings, materials like fly ash, silica, graphene oxides, and metallic oxides can also exhibit antimicrobial corrosion activities in concrete hybrid mixtures. Copper oxide, titanium oxides, nickel oxides, and oxides of aluminum and magnesium, which are known for their selective resistance against corrosion, can also be used to design antimicrobial concrete mixtures for longer-lasting concrete structures [1,50].

Despite the giant strides and improvement in microbial-induced corrosion resistance measures, their applications and implementation remain limited owing to several factors, such as the toxicity of the material or products formed, cost implications of some applications, and heterogeneity of most plastic mixtures, among others. It is, therefore, imperative to look for innovative, cost-effective, environmentally friendly, and a multipurpose solution to this MIC problem.

### 2.3. Current Approaches for Mitigation of MIC

**Figure 9:**
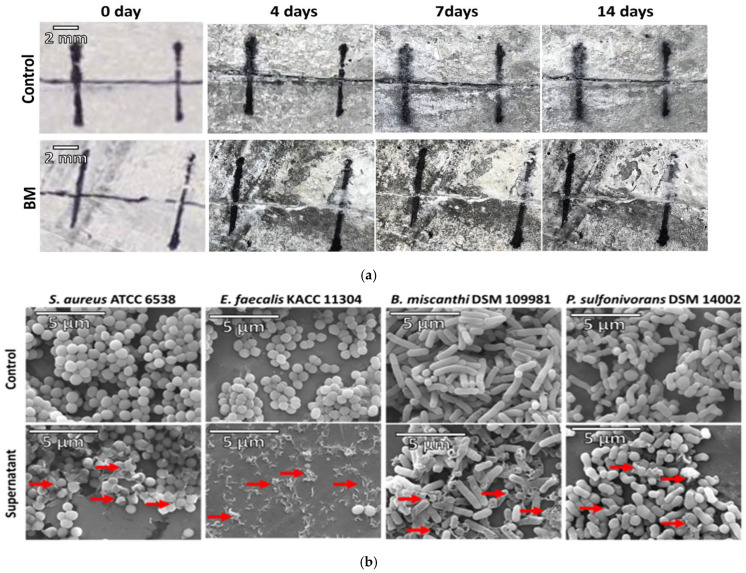

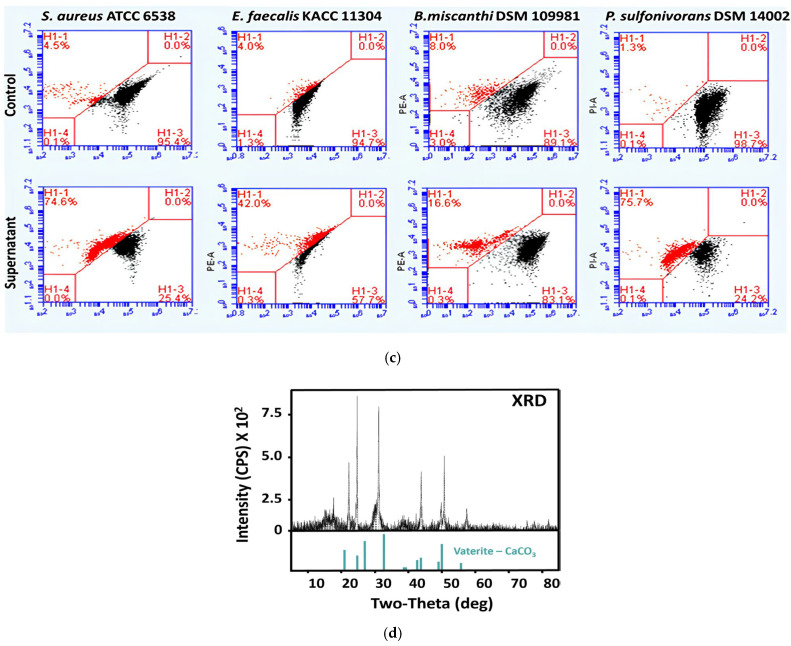
(**a**) Assessment of crack sealing in mortar as a function of curing time and crack width. Characterization and bactericidal effects of antibacterial substances produced by *B. altitudinis* B6 against four Gram-positive bacteria. The FE-SEM images reveal that cracks on the BM samples were completely healed within 14 days. (**b**) FE-SEM images of B6 strain phenotypes indicating the presence or absence of cell-free supernatant treatment. The red arrows show signs of cell death, including cell wall destruction and the presence of cellular debris. (**c**) Results of the live/dead cell viability assay, stained with fluorescence dyes (SYTO 9, propidium iodide), with flow cytometer analysis for validating the killing effects. (**d**) XRD analyses of CaCO_3_ precipitation in BM. The XRD results confirmed that the CaCO_3_ minerals (the whitish minerals seen on (**a**)) are vaterite. (BM: mortar treated with the B6 strain; Control: control mortar). Reproduced with permission from Min et al. [80]. © 2024 Elsevier Ltd., Amsterdam, The Netherlands.

The use of biocides (e.g., HOCl, ozone, NH_2_Cl, etc.) and surface coating remain the widely used approaches for MIC mitigation. These approaches have been helpful to a large extent; however, their long-term use and environmental impact are being put into question [75]. In recent years, research attention has been focused on the development of bacteria-based self-healing cementitious materials with antibacterial properties [76,77]. The general idea is to develop materials where the microcracks are sealed via bio-mineralization (e.g., precipitation of carbonate, a process commonly referred to as microbiologically induced calcium carbonate precipitation (MICP)) [78]. MICP was proposed in 2010 by Jonkers et al. [79] when alkali-resistant spore-forming bacteria related to the genus *Bacillus* were directly added to cement-based materials. The sealing up of microcracks prevents the growth of pathogenic bacterial communities on concrete surfaces and in extension MIC [80]. Min et al. [81] isolated a non-ureolytic and alkali-tolerant B6 strain from paddy soil designated as *Bacillus altitudinis* and studied its ability to induce carbonate precipitation and antibacterial activity against pathogens. Field emission scanning electron microscopy (FE-SEM) and X-ray diffractometry (XRD) results disclosed that the strain formed vaterite and produced extracellular polymeric substances that enhanced biofilm formation, even under high pH conditions. The authors noted that strain *B. altitudinis* B6 resulted in MICP, which repaired 0.3 mm microcracks within 14 days (Figure 9a), as well as produced antibacterial substances that could damage the cellular membranes of Gram-positive bacteria, including *Staphylococcus aureus* and *Enterococcus faecalis* (Figure 9b,c). Son et al. [76] investigated the CaCO_3_ precipitation performance of ureolytic and non-ureolytic bacteria co-cultured as a self-healing agent for cementitious materials’ crack repair and also studied the influence of coculturing ureolytic and non-ureolytic bacteria on microbial metabolism by measuring the rate of growth in urea-containing medium and the rate of NH_4_^+^ and CaCO_3_ production in urea–calcium lactate medium. It was found that CaCO_3_ precipitation is improved by co-culturing ureolytic and non-ureolytic bacteria, owing to the relatively faster growth rate of non-ureolytic bacteria. Several other pieces of research have been devoted to MICP studies [77,78] as well as the effect of key parameters such as pH, temperature, and the application method (direct or indirect addition) [77], but very recent works are geared toward understanding the formation and structure of MICP products [81].

Based on the promising potential of microorganisms to repair local structural defects in concrete, as discussed in the previous paragraph, it is obvious that the biogenic corrosion mitigation technique is the way forward. Hence, the present paper briefly discusses four microbiome-inhibiting microbiologically influenced corrosion (MIMIC) techniques: biological competition, secretion of antibiotic substances, inhibition of microbial attachment, and phagocytosis of microorganisms. These techniques were selected due to the environmental friendliness observed during their preliminary use in oil and gas wells for MIC control. Based on the advantages already presented by them in the oil and gas sector, these four MIMIC techniques are expected to be extended to concrete MIC control in the future.

#### 2.3.1. Biological Competition

**Figure 10:**
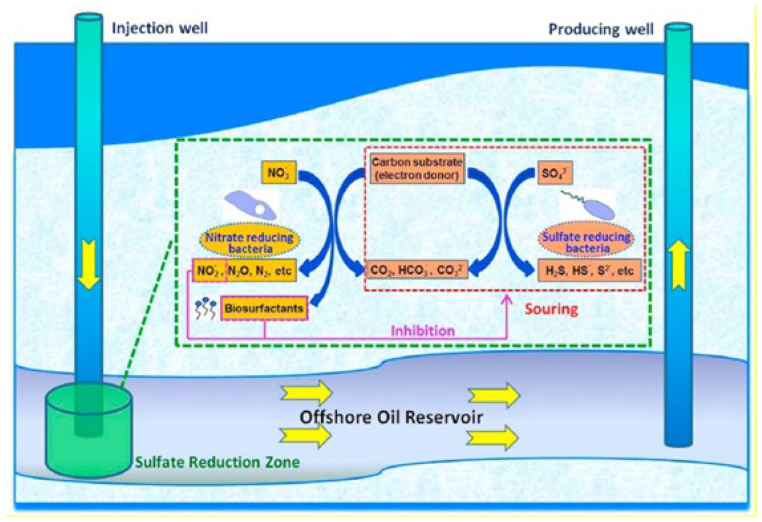
A schematic diagram illustrating the interaction of NRB and SRB in a souring environment. The mechanism of nitrate injection includes the augmentation of heterotrophic nitrate-reducing bacteria to outcompete SRB for available nutrients, promotion of nitrate-reducing sulfide-oxidizing bacteria to directly oxidize sulfide, and SRB inhibition through the resultant nitrite. The yellow arrows illustrate the biosurfactant injection and the prolonged effective duration of nitrate that helped nitrate/nitrite reach deeper zone in the system, resulting in long-term suppression of SRB activities and better souring control. Reproduced with permission from Fan et al. [82]. © 2019 Elsevier Ltd., Amsterdam, The Netherlands.

The biological competition approach takes advantage of the fact that microbial communities can communicate with each other by releasing signals. Such communication can either foster synergistic metabolism or induce competitive metabolism. The technique is designed to induce competitive metabolism between beneficial microorganisms and pathogenic ones. For instance, injecting nitrate into an oil well is a promising strategy in the remediation of H_2_S corrosion. In an SRB-induced sour well, the injection of nitrate into the well boosts the metabolic ability of heterotrophic nitrate-reducing bacteria (hNRB) to outcompete with SRB (bio-competitive exclusion) for the degradation of organics and also encourages the nitrate-reducing sulfide-oxidizing bacteria (NR-SOB) to directly oxidize sulfide [82]. More so, depending on the temperature, NRB reduces nitrate to nitrite and further to nitrogen gas [82]. Nitrite can inhibit sulfide formation (souring) by SRB [83,84]. Hubert and Voordouw [83] reported that the souring of a bioreactor containing 12.5 mM lactate and 6, 2, or 0.75 mM sulfate can be mitigated by the injection of 10 mM nitrate. As previously mentioned, the success of the nitrate injection strategy depends to a large extent on the temperature of the system. Fida et al. [84] reported that nitrite accumulation can be achieved at temperatures ranging from 50 to 70 °C. In the investigation of Fan et al. [82] on the use of NRB (*Thauera*, *Pseudomonas*, *Petrobacter*, *and Geobacillus*) to inhibit sour corrosion induced by the SRB strain, *Desulfomicrobium escambiense* (Figure 10), it was observed that the NRB reduced nitrate to nitrite and nitrite to N_2_ at temperatures ≤ 45 °C. Above 45 °C, the bacteria were unable to further reduce nitrite to N_2_.

#### 2.3.2. Secretion of Antibiotic Substances

Recent studies have shown that some bacteria can secrete inhibitors or antimicrobial substances that inhibit the growth of other bacteria. Korenblum et al. [85] isolated forty bacteria strains from a Brazilian oil reservoir and tested them against each other. It was found that the strains *Bacillus subtilis* (LFE-1), *Bacillus firmus* (H_2_O-1), and *Bacillus licheniformis* (T6-5) produced inhibitory substances that were stable over a wide range of pH and temperature values and were able to suppress the growth of 65% of other Bacillus strains. The strains T6-5 and H_2_O-1 were found to be active against SRB. In a following study, Korenblum et al. [86] grew strain T6lab (i.e., an oil reservoir SRB strain) in addition to the *Bacillus* strains previously studied (i.e., LFE-1, H_2_O-1, and T6-5). The biofilms of these four strains were grown on glass surfaces to demonstrate the efficacy of the produced inhibitory substances by the *Bacillus* strains. It was found that the inhibitory substance produced by T6-5 and H_2_O-1 could abort the biofilm formation by *B. pumilus*, as well as eradicate the pre-grown biofilm of *B. pumilus*. The inhibitory substance by H_2_O-1, on the other hand, was found to be potent in reducing the viability and adherence of SRB consortium biofilm, demonstrating the promising future of using these naturally inhibiting substances for MIC mitigation.

#### 2.3.3. Phagocytosis of Microorganisms

A bacteriophage or phagocyte is a kind of bacterium that preys on other bacteria. The unique characteristics of bacteriophage are specificity, self-replication, and exponential proliferation. Qiu et al. [87] reported that *B. dellovibrio bacteriovorus*, a Gram-negative bacterium, can be used to inhibit SRB-induced MIC. The authors found that *B. bacteriovorus* retarded the growth of SRB by its predatory action. Results from electrochemical impedance spectroscopy showed that *B. bacteriovorus* inhibited SRB-induced corrosion of the X70 pipeline. Qiu et al. [87] observed that the average corrosion rate of the X70 pipeline steel coupons reached up to 19.17 mg·dm^−2^·day^−1^ after 60 days of exposure to medium with SRB, while the inoculation of *B. bacteriovorus* brought down the corrosion rate to 3.75 mg·dm^−2^·day^−1^. However, the disadvantage of this technique is its high specificity for phagosomal enzymes—a fact that may delay its widespread adoption. In nature, mixed-strain biofilms are more common than single-strain biofilms.

#### 2.3.4. Inhibition of Microbial Attachment

The inhibition mechanism through microbial attachment suppression is related to quorum sensing (QS), a cell-to-cell communication process. As mentioned earlier, microorganisms can produce signal molecules that release some specific self-inducing substances, transmit them into the cell through signal transmission, affect the expression of specific genes, and regulate the physiological characteristics of the microbial population [88]. QS permits a bacterial population to mount a cooperative response that improves access to nutrients, promotes defense against competitors, as well as survives in adverse environmental conditions [89]. QS inhibitors thus interfere with bacterial QS systems and block the information communication within or between populations. The lichen secondary metabolite evernic acid and the carotenoid zeaxanthin are effective QS inhibitors against the expression of *P. aeruginosa*, with zeaxanthin showing greater efficacy [89]. Gökalsın et al. [90] used carotenoid zeaxanthin to reduce the expressions of *P. aeruginosa* virulence factors through quorum sensing inhibition. The inhibition potential of zeaxanthin was assessed through in silico screening from a library of 638 lichen metabolites. Fluorescent monitor strains were used for quorum-sensing inhibitor screens, and the quantitative reverse-transcriptase PCR assay was used for the evaluation of gene expression. It was found that zeaxanthin exhibited a better inhibitory effect than the lichen secondary metabolite evernic acid, which previous works [90] showed to exhibit an inhibiting effect against *P. aeruginosa* quorum-sensing systems.

Although these four MIMIC techniques are still very much at the laboratory stage, because the principle is based on the utilization of non-pathogenic microorganisms for MIC control and microorganisms are available everywhere, MIMIC might be the way forward in terms of long-term and environmentally friendly MIC mitigation strategies.

## 3. Conclusions

The metabolic activity of microorganisms on the surface of a metal can influence MIC. Their activity can directly influence the redox reactions and/or create an aggressive environment that leads to surface degradation. Some common MIC microorganisms are: SRB, which feeds on sulphates and excretes H_2_S; SOB, which feeds on sulfur and excretes H_2_SO_4_; and NRB, which converts nitrate to nitrite and then to N_2_. The traditional ways of controlling MIC include the application of biocides and material modification. Some of the traditional techniques are not environmentally friendly; so, new methods are needed. One of the modern methods discussed in this paper involves using bacteria-based self-healing cementitious materials with antibacterial activity. These materials prevent the attachment and growth of pathogens on the surface of the asset, and, by extension, retard MIC. The present paper also discusses four microbiome-inhibiting microbiologically influenced corrosion techniques, namely biological competition, the secretion of antibiotic substances, inhibition of microbial attachment, and phagocytosis of microorganisms, which have been experimented with in oil and gas wells for MIC control and may find future application in concrete MIC control. It should be mentioned that the MIMBIC techniques discussed in this paper are still at the laboratory level.
